# Identification and validation of BATF as a prognostic biomarker and regulator of immune cell infiltration in acute myeloid leukemia

**DOI:** 10.3389/fimmu.2024.1429855

**Published:** 2025-01-13

**Authors:** Zhe Zhao, Dongmei Wang, Xue Sheng, Shuying Li, Tingting Liu, Mengyuan Chang, Lei Feng, Di Zhang, Chunyan Ji, Fei Lu, Jingjing Ye

**Affiliations:** ^1^ Department of Hematology, Qilu Hospital of Shandong University, Jinan, China; ^2^ Shandong Key Laboratory of Hematological Diseases and Immune Microenvironment, Qilu Hospital of Shandong University, Jinan, Shandong, China

**Keywords:** BATF, immune cell infiltration, prognosis, proliferation, drug sensitivity, AML

## Abstract

**Background:**

Basic leucine zipper ATF-like transcription factor (BATF) is a nuclear basic leucine zipper protein affiliated with the AP-1/ATF superfamily. Previous research has confirmed that BATF expression plays a significant role in the tumour microenvironment. However, the associations between BATF expression and prognoses in acute myeloid leukaemia (AML) patients and their immunological effects remain unclear.

**Methods:**

Genomic and clinical AML data were got from the TCGA (TCGA-LAML) and GEO (GSE37642) databases for the subsequent analysis. The expression levels of BATF in AML patients were assessed using GEPIA, and the results were verified by qRT-PCR and Western blotting. In the meantime, the prognostic value of BATF was evaluated using univariate and multivariate analyses, receiver operating characteristic (ROC) curve (AUC) analysis, and Kaplan-Meier (KM) survival analysis. EdU, colony formation, and CCK-8 assays were employed to evaluate the proliferation of cells. Moreover, we detected the association of BATF expression with drug sensitivity through database analysis and *in vivo* experiments. To further investigate the mechanism of action of BATF in AML, RNA sequencing (RNA-seq) analysis was performed, followed by pathway enrichment analysis using Kyoto Encyclopedia of Genes and Genomes (KEGG) pathway analysis. Meanwhile, we detected the connection between BATF expression and the proportions of immune cells via flow cytometry in C1498 mouse model of AML. Finally, we investigated the association between BATF expression and cell-cell communication within the AML cell population using single-cell sequencing.

**Results:**

In this study, we thoroughly investigated the role of BATF in AML. First, we observed a significant elevation in the expression of BATF in patients and cells in AML. Further analysis revealed an association between high BATF expression and poor prognosis in AML. Additionally, BATF expression was found to promote the proliferative capacity of AML cells. Moreover, the results showed that the expression level of BATF dramatically affected the effect of chemotherapy in AML patients. We also discovered that BATF expression could activate multiple immune-related pathways, altering the proportions of CD8^+^T cells and NK cells, suggesting that BATF may be a regulator of immune cell infiltration. Finally, there were differences in receptor ligand pairs between AML cells with high and low expression of BATF and immune cells.

**Conclusion:**

Bioinformatics analysis and experimental verification revealed that BATF expression could alter the proportions of CD8^+^T cells and NK cells in AML and affect drug sensitivity, making it a potential treatment target for AML.

## 1 Introduction

AML is a malignant clonal disease characterised by aberrant proliferation and abnormal differentiation of myeloid blasts, resulting in abnormal haematopoietic function (1). The complete remission (CR) and 5-year disease-free survival (DFS) rates of AML patients have improved with the development of various therapeutic approaches, including combination chemotherapy, gene targeted therapy, and haematopoietic stem cell transplantation (2, 3). Nonetheless, 40-50% of AML patients relapse within a certain time, and their prognoses are generally poor (4, 5). Consequently, there is clinical significance for finding a novel target for AML therapy.

BATF is a nuclear basic leucine zipper protein in the activated protein-1 (AP-1) superfamily that binds to the Jun protein to induce BATF/Jun dimerisation to inhibit activity of the AP-1 pathway (6, 7). BATF family proteins dimerise with JUN family proteins and can recognise 7-long TPA response elements or 8-long cyclic AMP response elements (8). These proteins, composed of 125 amino acids, solely possess the bZIP domain without any additional domains. Previous researches have shown that BATF participates in the development and functionality of T helper (Th) cells, including Th2, Th9, Th17, and follicular Th cells (9–12), and is indispensable for the initial activation of CD8^+^-T cells (13). Yao et al. indicated that BATF could control the transformation of progenitor to cytolytic effector CD8^+^T cells in the event of a chronic viral infection (14). Moreover, BATF sustains the functions of tissue-resident Treg cells (15, 16), which supports the therapeutic utilisation of tissue Treg cells’ regeneration abilities.

Recent studies have shown that across several disorders, BATF is associated with macrophage activation and cancer growth. BATF has been demonstrated to enhance the antitumour response of CAR-T cells (17). Moreover, Feng et al. indicated that BATF also acts as a cancer-promoting gene in lung cancer (18). Furthermore, Schleussner et al. confirmed that inactivation of BATF or inhibition of AP-1 resulted in ALK^+^ and ALK^-^ ALCL growth hindrance and/or cell death (19). Additionally, an 8-gene signature-based risk score model including the BATF gene has been constructed and shown to correlate with prognosis in AML patients (20). Nonetheless, the fundamental molecular mechanisms underlying the potential role of BATF in AML development remain elusive.

Here, we used bioinformatics analysis and experiments to examine the impact of BATF on AML. We identified that BATF was highly expressed and connected to a poor prognosis in AML patients. *In vitro* assays indicated that BATF promoted cell proliferation. Next, an analysis and experimental verification of drug sensitivity were performed to investigate the correlation between BATF expression and clinical therapeutic outcomes. Furthermore, RNA-seq analysis and *in vivo* experiments revealed that BATF had a remarkable immunological regulatory effect on modifing the the proportions of immune cells. In conclusion, the present findings suggest that BATF could alter the AML immune cell infiltration and may be an effective treatment target for AML.

## 2 Materials and methods

### 2.1 Data collection

The TCGA database contains publicly available data on transcriptome matrices and clinical material and was downloaded for study. A Perl script was used to extract and integrate the transcriptomic matrices for each AML sample, which were subsequently used as a training set. The GSE37642 dataset from the GEO database was utilised as the validation set. Additional gene expression data from normal tissues were obtained from the GTEx database. **Table 1** displayed the baseline characteristics of the AML patients who were included in the analysis. Correlation analysis was performed through LinkedOmics. In this study, we used the GEPIA to perform a prognostic analysis of BATF expression and differential mRNA expression in AML patients and healthy controls. 0.05 was chosen for the *P* value significance cut-off.

**Table 1:** Association analysis between BATF expression levels and clinicopathologic features in the TCGA-LAML database.

Variable	Low expression of BATF	High expression of BATF	*P*
n	76	75	
Gender, n (%)			0.455
Female	31 (20.5%)	36 (23.8%)	
Male	44 (29.1%)	40 (26.5%)	
Race, n (%)			0.714
Asian	1 (0.7%)	1 (0.7%)	
Black or African American	5 (3.3%)	8 (5.3%)	
White	68 (45.3%)	67 (44.7%)	
Age, n (%)			0.060
≤60	48 (31.8%)	36 (23.8%)	
>60	27 (17.9%)	40 (26.5%)	
WBC count(х10^9^/L), n (%)			0.869
≤20	38 (25.3%)	38 (25.3%)	
>20	36 (24.0%)	38 (25.3%)	
BM blasts (%), n (%)			0.663
≤20	28 (18.5%)	31 (20.5%)	
>20	47 (31.1%)	45 (29.8%)	
PB blasts (%), n (%)			0.572
≤70	37 (24.5%)	34 (22.5%)	
>70	38 (25.2%)	42 (27.8%)	
Cytogenetic risk, n (%)			0.001
Poor	15 (10.1%)	21 (14.1%)	
Intermediate	34 (22.8%)	49 (32.9%)	
Favorable	24 (16.1%)	6 (4.0%)	
FAB classification, n (%)			0.001
M0	10 (6.7%)	5 (3.4%)	
M1	17 (11.4%)	18 (12.1%)	
M2	18 (12.1%)	20 (13.4%)	
M3	13 (8.7%)	1 (0.7%)	
M4	13 (8.7%)	16 (10.7%)	
M5	2 (1.3%)	13 (8.7%)	
M6	0 (0.0%)	2 (1.3%)	
M7	1 (0.7%)	0 (0.0%)	
FLT3 mutation, n (%)			0.701
Negative	46 (32.6%)	53 (37.6%)	
Positive	21 (14.9%)	21 (14.9%)	
IDH1 R132 mutation, n (%)			0.174
Negative	60 (42.3%)	70 (49.3%)	
Positive	8 (5.6%)	4 (2.8%)	
IDH1 R172 mutation, n (%)			0.960
Negative	68 (47.6%)	73 (51.0%)	
Positive	1 (0.7%)	1 (0.7%)	
IDH1 R140 mutation, n (%)			0.465
Negative	62 (43.4%)	69 (48.3%)	
Positive	7 (4.9%)	5 (3.5%)	
RAS mutation, n (%)			0.903
Negative	65 (45.1%)	71 (49.3%)	
Positive	4 (2.8%)	4 (2.8%)	
NPM1 mutation, n (%)			0.130
Negative	57 (39.6%)	54 (37.5%)	
Positive	12 (8.3%)	21 (14.6%)	

### 2.2 RNA sequencing analyses

Bulk RNA-seq analysis was conducted using total RNA extracted from C1498-Control and C1498-shBATF cells. The “limma” package was used to identify differentially DEGs in the low- and high-expression BATF groups with thresholds set to |fold change| ≥ 1 and *P* values < 0.05. Using the “clusterProfiler” R package, KEGG analyses were used to enrich DEGs in biological processes and signalling pathways.

### 2.3 Prognostic gene expression and clinical statistical analysis

The R package “survival” employed a log-ranking algorithm to generate Kaplan-Meier survival curves, which were then utilised to evaluate the OS rates of AML patients in the low- and high-expression groups. The Kaplan-Meier survival curves of GSE6891 was obtained from Kaplan-Meier plotter website. We also analysed the univariate Cox regression analysis between survival and multivariate variables. The “timeROC” packages was used for ROC curve analysis. The relationship between BATF expression and clinical characteristics was assessed through log-rank tests. This study investigated how varying levels of BATF expression, categorized as high or low, correlated with different clinical features.

### 2.4 Immune checkpoint genes and drug sensitivity analysis

The immune checkpoint genes of BATF were identified from the gene expression data, and the correlations between these genes were calculated. Based on the GDSC database, antitumour drug response was predicted by the R package “OncoPredict” for each AML patient with high and low expression. Treatment group C57BL/6 mice were treated with cytarabine (MCE) as an anti-cancer chemotherapy drug at 50 mg/kg via intraperitoneal injection for up to 2 weeks, while venetoclax (MCE) at 100 mg/kg via oral gavage daily for up to 1 weeks.

### 2.5 Cell culture

Seven AML cell lines (THP-1, MOLM-13, Kasumi-1, HL-60, KG-1, OCI-AML3, and C1498) and HEK 293T cells were obtained from the ATCC. THP-1 and MOLM-13 cells were routinely cultured in RPMI-1640 (Gibco) with 10% FBS (Gibco). Kasumi-1, HL-60 and KG-1 cells were cultured in IMDM (Gibco) supplemented with 20% FBS. OCI-AML3 cells were cultured in RPMI-1640 supplemented with 20% FBS, while HEK 293T and C1498 cells were grown in DMEM (Gibco) supplemented with 10% FBS.

### 2.6 RNA isolation and quantitative RT-PCR

To extract total RNA, TRIzol reagent from ACCURATE Biotechnology Co., Ltd. was utilized. After the RNA concentration was measured, reverse transcription was carried out utilizing HiScript II Q RT SuperMix, specifically designed for qPCR applications, sourced from Vazyme. The expression levels of RNA were assayed through the employment of SYBR Green reagents (Vazyme) in conjunction with suitable primers. The primer sequences utilized in this study are presented in **Supplementary Table S1**.

### 2.7 Western blotting

Extraction of proteins was accomplished using M-PER™ Mammalian Protein Extraction Reagent from Thermo Scientific, which contained protease inhibitors and phosphatase inhibitors, both sourced from Roche. The proteins were resolved by SDS-PAGE and subsequently transferred onto a PVDF membrane from Millipore (USA). Prior to incubation with antibodies, the PVDF membrane was blocked with 5% milk dissolved in TBST at room temperature for 1 hour. Following blocking, the membrane was incubated with primary antibodies and subsequently with HRP-conjugated secondary antibodies. The results were detected by a chemiluminescent imaging system (Champchemi). Primary antibodies targeting BATF were obtained from CST. Anti-GAPDH (AB0038) was purchased from Abways Technology.

### 2.8 Plasmid construction and retroviral transfection

The BATF overexpression plasmid was purchased from Shandong Shuo Bo Yun Biotechnology Co., Ltd (China). We constructed a BATF-shRNA plasmid by using the PLKO.1-TRC cloning vector as the empty plasmid. Plasmid recombination was utilised to generate the BATF plasmid with some specified portions silenced (experimental group). The shRNA sequence was synthesised by MERCK. The sequence of the BATF shRNA is detailed in **Supplementary Table S2**. The packaging and envelope plasmids used for virus synthesis were psPAX2 and pMD2.G. We transduced lentiviruses harvested from HEK 293T cells transfected with pLKO.1-puro or shBATF. To transduce cells, they were infected with lentivirus, and those cells successfully transduced were then selected using puromycin.

### 2.9 Proliferation

To evaluate cell proliferation using the CCK-8 assay, cells were inoculated in 96-well plates and incubated at 37°C in a controlled environment. 10 μL of CCK-8 reagent (Vazyme Biotech, Nanjing, China) was added to wells at certain times. Using a microplate reader from BioTek (USA), the absorbance at 450 nm was accurately measured.

To assess proliferation by colony formation assay, cells were incubated in methylcellulose medium (MethoCult™ H4230, Stem Cell Technologies) in accordance with the manufacturer’s recommended protocol. Colonies were imaged after 10 days of incubation.

To assess proliferation by the EDU assay, the cells were plated in 12-well plates, and subsequently incubated with the EDU working solution for a duration of 4 hours. The cells were then fixed and stained adhering to the manufacturer’s specified guidelines (RiboBio, Guangzhou, China) and photographed using a Zeiss confocal microscope (LSM 900).

### 2.10 Purification of immune cells and flow cytometry analysis

To examine the composition of immune cells within the tumor immune microenvironment (TIME), mononuclear cells from the bone marrow and spleen were collected after 24 days post-inoculation for flow cytometry analysis. Meanwhile, immune cell proportions in the spleen and bone marrow were evaluated. Cells were stained with flow cytometry antibodies and incubated at 37°C for 30 minutes. All flow cytometry antibodies used in the study were obtained from BioLegend: PerCP/Cyanine5.5 anti-mouse CD3 (17A2), PE/Cyanine7 anti-mouse CD8a (53-6.7), APC anti-mouse CD4 (RM4-5), PE anti-mouse F4/80 (W20065B), APC anti-mouse CD206 (C068C2), PE/Cyanine7 anti-mouse CD11b (M1/70), APC anti-mouse Gr-1 (R86-8C5), APC anti-mouse NK-1.1 (PK136), and APC anti-human CD274 (PD-L1) (29E.2A3).

### 2.11 Murine model

C57BL/6 mice (female, 6-8 weeks old) were obtained from Beijing Vital River Laboratory Animal Technology. To establish the AML mouse model, C1498 cells transfected with GFP (10^6^ cells/host) were injected into the mice via the tail vein. After 24 days, the mice were euthanized, and mononuclear cells from the bone marrow and spleen were collected. Flow cytometry was used to assess the percentage of GFP^+^ cells among the mononuclear cell population. Besides, heart, liver, spleen, lung and kidney samples of mice were collected for HE staining. Male NOG mice (6-8 weeks old; Vital River Laboratory Animal Technology, Beijing) were intravenously injected with GFP transfected C1498 control cells or C1498 shBATF cells. All animal procedures were approved by the Animal Ethics Committee of Qilu Hospital, Shandong University.

### 2.12 Patients and clinical samples

Bone marrow samples were collected from 32 patients with AML and 16 healthy controls at the Qilu Hospital of Shandong University in China. The average age of AML patients was 64.50 years, while the average age of healthy controls was 60.56 years. Mononuclear cells were isolated from bone marrow samples using Ficoll density gradient centrifugation. CD34^+^ cells were isolated using CD34 MicroBeads following the protocol provided by the manufacturer (Miltenyi Biotec). The Medical Ethics Committee of Qilu Hospital, Shandong University (KYLL-202111-022-1), approved all laboratory experiments on primary samples. Informed consent was obtained following the Helsinki Declaration and national laws.

### 2.13 Single- cell sequencing analysis

The GSE116256 and GSE255245 dataset from the GEO database was utilised in the single-cell sequencing analysis. Cell-cell interaction analysis focused on the specific interactions between malignant cells, classified by high or low BATF expression, and other cell types within various receptor-ligand pairs. The results were then analyzed and visualized to illustrate these interactions.

### 2.14 Statistical analysis

R software (version 4.2.2), Perl scripts and GraphPad Prism 9 software were used for the statistical analyses in this study. *P*<0.05 was regarded as statistically significant.

## 3 Results

### 3.1 BATF is highly expressed and associated with poor prognosis in AML patients

**Figure 1:**
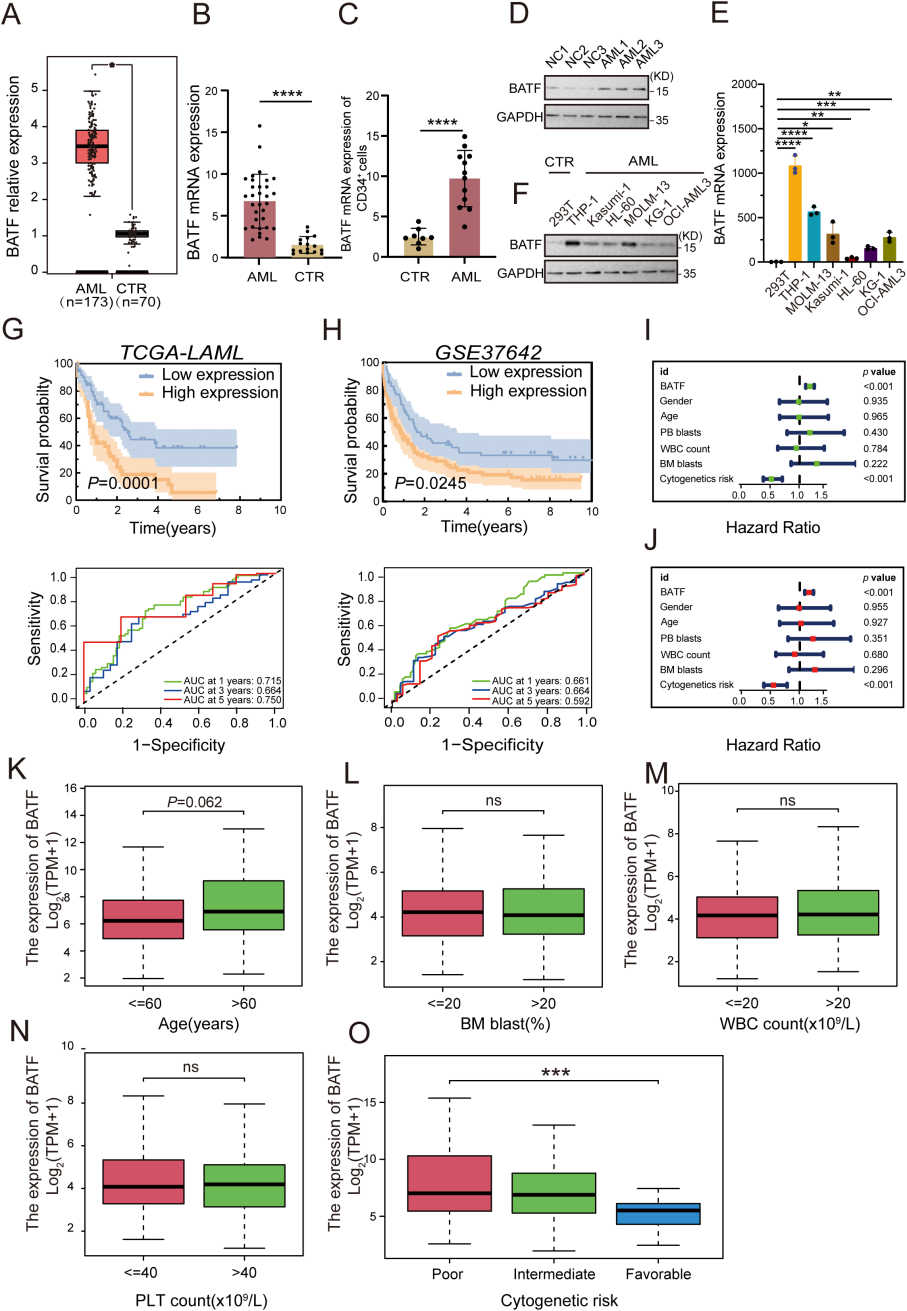
BATF exhibits a high level of expression in AML patients, which is inversely associated with their overall survival rates. **(A)** BATF expression levels in AML patients (n=173) and normal controls (n=70) from the GEPIA database. **(B)** BATF mRNA expression in AML patients (n=32) and normal controls (n=16) was detected by qRT-PCR. **(C)** qRT-PCR was used to analyze BATF mRNA expression in CD34^+^ leukemia cells isolated from AML patients (n=12) and healthy controls (n=8). **(D)** Western blotting analysis of BATF expression in patients with AML (n = 3) and control patients (n = 3). **(E)** qRT-PCR analysis of BATF expression in leukaemia cells compared to that in control cells. **(F)** Western blotting analysis of BATF expression in AML cell lines relative to that in control cells. **(G)** KM survival analysis and the AUC of BATF in the TCGA database. **(H)** KM survival analysis and the AUC of BATF in the GEO database. **(I, J)** Univariate and multivariate analyses of BATF in the TCGA database. **(K–O)** BATF expression levels in age, BM blasts, WBC count, PLT count and cytogenetic risk subgroups according to the TCGA database. *P < 0.05, **P < 0.01, ***P < 0.001, ****P < 0.0001, ns, nonsignificant.

To detect the expression of BATF, the GEPIA database was used to compare its expression levels between AML patients and healthy controls, revealing a significant elevation of BATF expression in AML samples (**Figure 1A**). Additionally, we detected the BATF expression in our clinical samples from AML patients and healthy controls and proved that BATF was highly expressed in AML patients (**Figures 1B–D**). In addition, the qRT-PCR and Western blotting results also showed that BATF was overexpressed in leukaemia cell lines compared to control cells, especially in the THP-1 and MOLM-13 cell lines (**Figures 1E, F**). Therefore, these two cell lines were chosen for subsequent studies. Moreover, our findings indicated that individuals within the high BATF expression group demonstrated a poorer prognostic outcome, according to the results of the KM survival analysis and the AUC of the two groups in the TCGA cohort (**Figure 1G**). In addition, for additional validation, the GEO dataset GSE37642 was used, and the results were identical to those of the TCGA (**Figure 1H**). Meanwhile, to further validate the relationship between BATF expression and survival in AML, we used the GSE6891 dataset to confirm the above conclusion (**Supplementary Figure S1A**). Furthermore, we stratified patients into younger and older groups based on age and assessed the survival impact of BATF expression. The results indicated that high BATF expression consistently correlated with poorer survival outcomes in both age groups (**Supplementary Figures S1B, C**). Similarly, we analyzed the correlation between BATF expression and overall survival in poor, intermediate, and favorable ELN risk groups. High BATF expression was significantly associated with worse survival in intermediate groups (**Supplementary Figures S1D–F**), suggesting that BATF expression might serve as a prognostic biomarker for AML patients. Univariate and multivariate analyses revealed that BATF was an independent risk factor for AML (**Figures 1I, J**). At the same time, we also found that the AUC value of BATF is higher than that of some other previously reported prognostic markers in TCGA-LAML database, such as RUNX1, CEBPA and FLT3 (**Supplementary Figures S1G–I**). To explore the correlation between BATF gene expression and clinical features, we integrated age, cytogenetic risk, white blood cell (WBC) count, platelet (PLT) count, and bone marrow (BM) blast percentages for visualization (**Figures 1K–O**). Analysis of the study data revealed that high BATF expression levels significantly differed from low BATF levels in terms of cytogenetic risk. Finally, we found that BATF related molecules were also highly expressed and had poor prognosis in AML, which further confirms our conclusion in **Supplementary Figure S2**. Overall, these data indicate that BATF is overexpressed in AML patients and exhibits a negative association with overall survival.

### 3.2 BATF promotes the proliferation of AML cells

**Figure 2:**
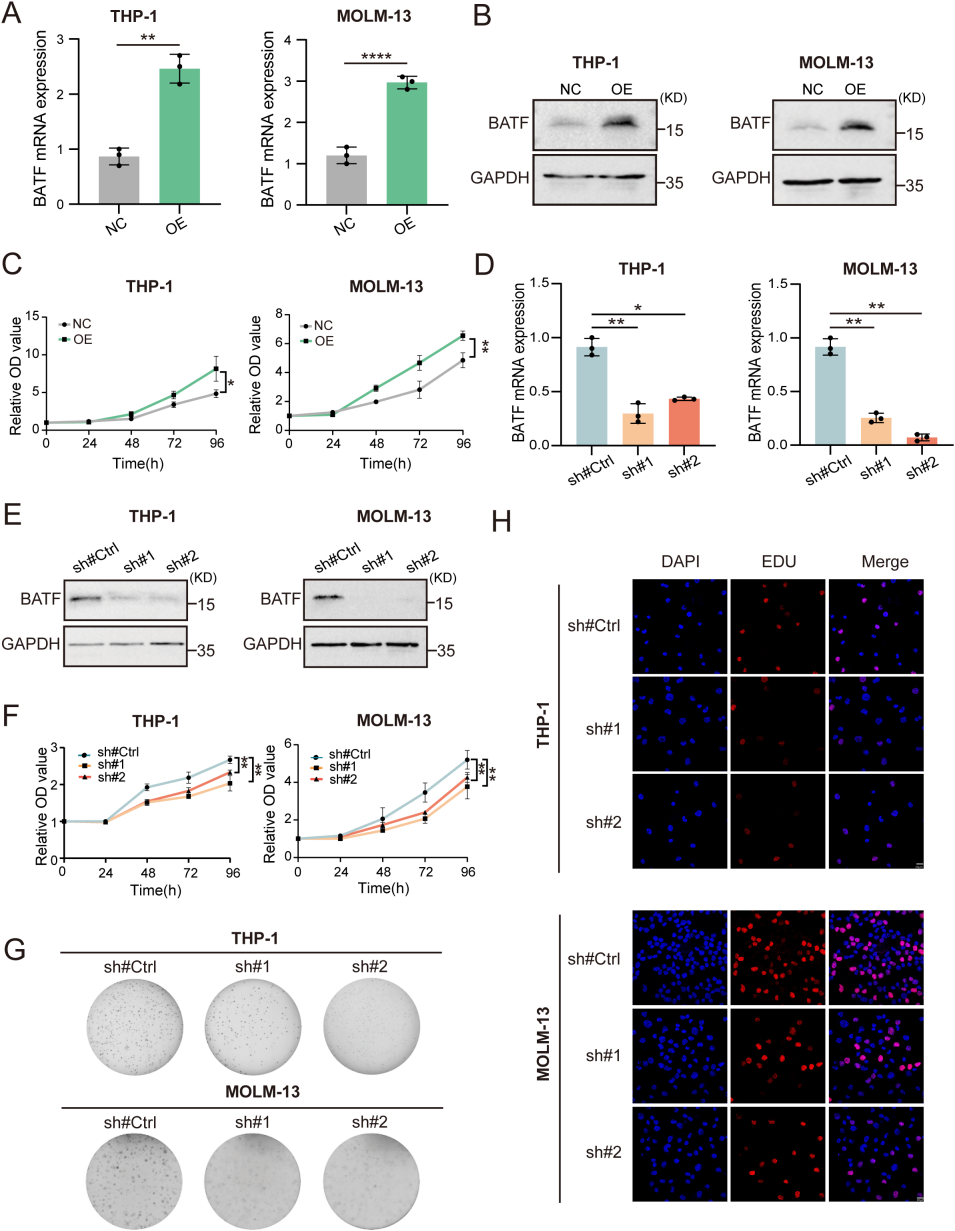
BATF expression stimulates the growth and multiplication of AML cells. **(A, B)** THP-1 and MOLM-13 cells were transduced with BATF-expressing lentivirus or control lentivirus. The overexpression efficacy of BATF was determined by qRT-PCR and Western blotting. **(C)** CCK-8 assay of BATF-overexpressing and control cells. **(D, E)** THP-1 and MOLM-13 cells were transduced with shBATF lentivirus or control lentivirus. The knockdown efficacy of BATF was determined by qRT-PCR and Western blotting. **(F)** CCK-8 assay of shBATF-treated and control cells. **(G)** Colony-forming ability of shBATF-treated and control cells. **(H)** EdU assay of shBATF and control cells. *P < 0.05, **P < 0.01, ****P < 0.0001.

To explore the impacts of BATF expression on cellular proliferation, we used a BATF-expressing lentivirus to overexpress BATF in THP-1, MOLM-13 and KG-1 cells (**Figures 2A, B**, **Supplementary Figure S3A**). A CCK-8 assay demonstrated that overexpression of BATF significantly promoted AML proliferation in both high BATF-expressing and low BATF-expressing AML cells (**Figure 2C**, **Supplementary Figure S3B**). Moreover, we used BATF-targeting shRNA lentivirus to decrease the expression of BATF (**Figures 2D, E**), and the proliferative capacity of THP-1 and MOLM-13 cells was obviously inhibited according to a CCK-8 assay (**Figure 2F**). In addition, a colony formation assay confirmed the same pattern (**Figure 2G**). Additionally, a lower proportion of EdU-positive cells was observed among the shBATF cells (**Figure 2H**). Collectively, these results confirmed that BATF expression stimulates the growth of AML cells.

### 3.3 Association of BATF expression with drug sensitivity

**Figure 3:**
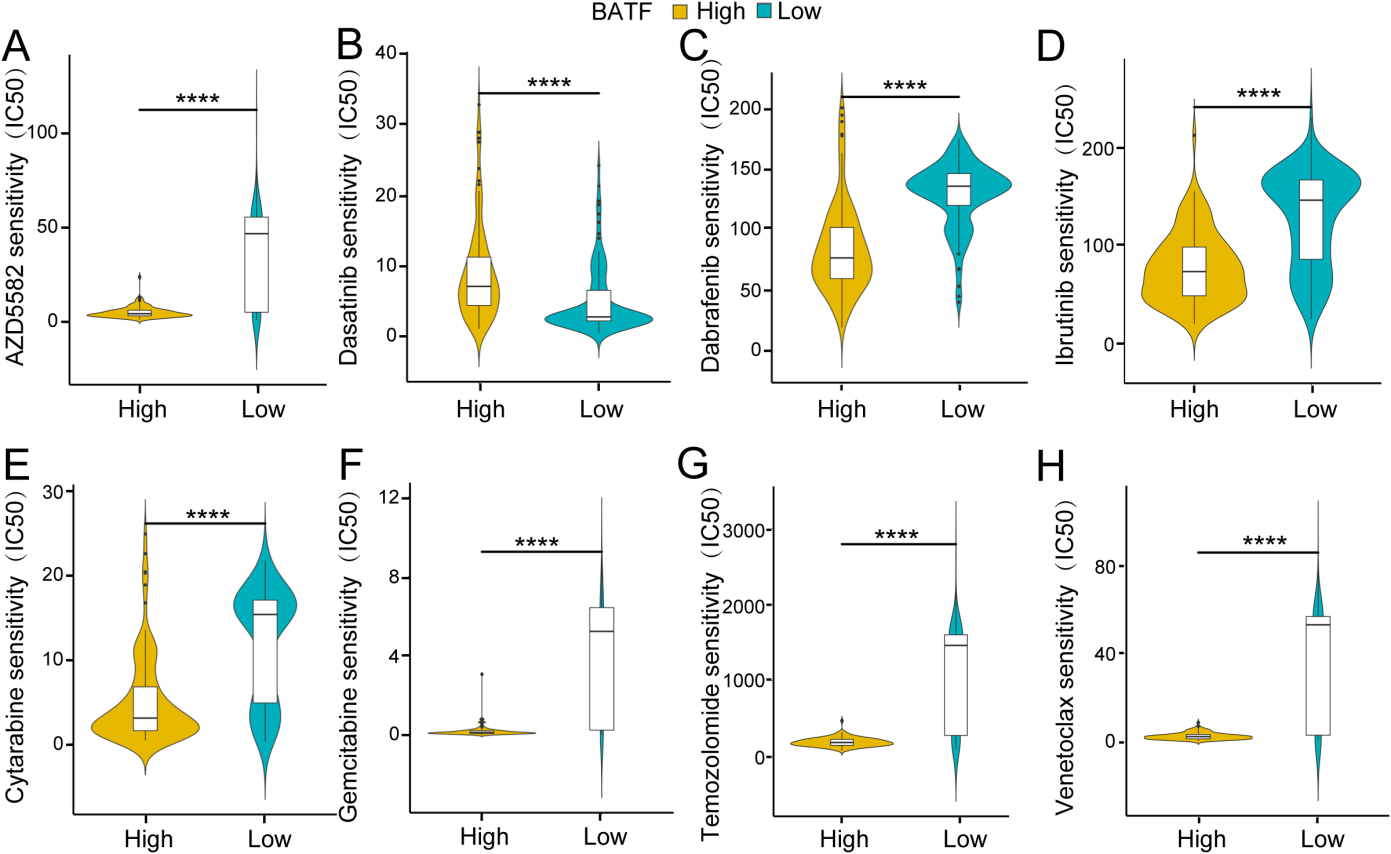
Association of the BATF expression level with drug sensitivity. **(A)** Association of BATF expression with AZD5582 sensitivity. **(B)** Association of BATF expression with Dasatinib sensitivity. **(C)** Association of BATF expression with Dabrafenib sensitivity. **(D)** Association of BATF expression with Ibrutinib sensitivity. **(E)** Association of BATF expression with Cytarabine sensitivity. **(F)** Association of BATF expression with Gemcitabine sensitivity. **(G)** Association of BATF expression with Temozolomide sensitivity. **(H)** Association of BATF expression with Venetoclax sensitivity. ****P < 0.0001.

Vital strategies in the clinical management of AML include chemotherapy and targeted therapy. Subsequent analysis revealed several potential antitumour agents that may be beneficial for the treatment of AML. As shown in **Figure 3**, the findings from the drug sensitivity analysis revealed that the IC50s of AZD5582, dabrafenib, ibrutinib, cytarabine, gemcitabine, temozolomide and venetoclax were significantly lower in the high-expression group, while that of dasatinib was greater in the high-expression group. The findings suggested that the expression level of BATF dramatically affected the effect of chemotherapy in patients with AML, providing new insights into precision therapy for AML patients.

### 3.4 The efficacy and side effects of cytarabine and venetoclax were analyzed in relation to BATF expression *in vivo*

**Figure 4:**
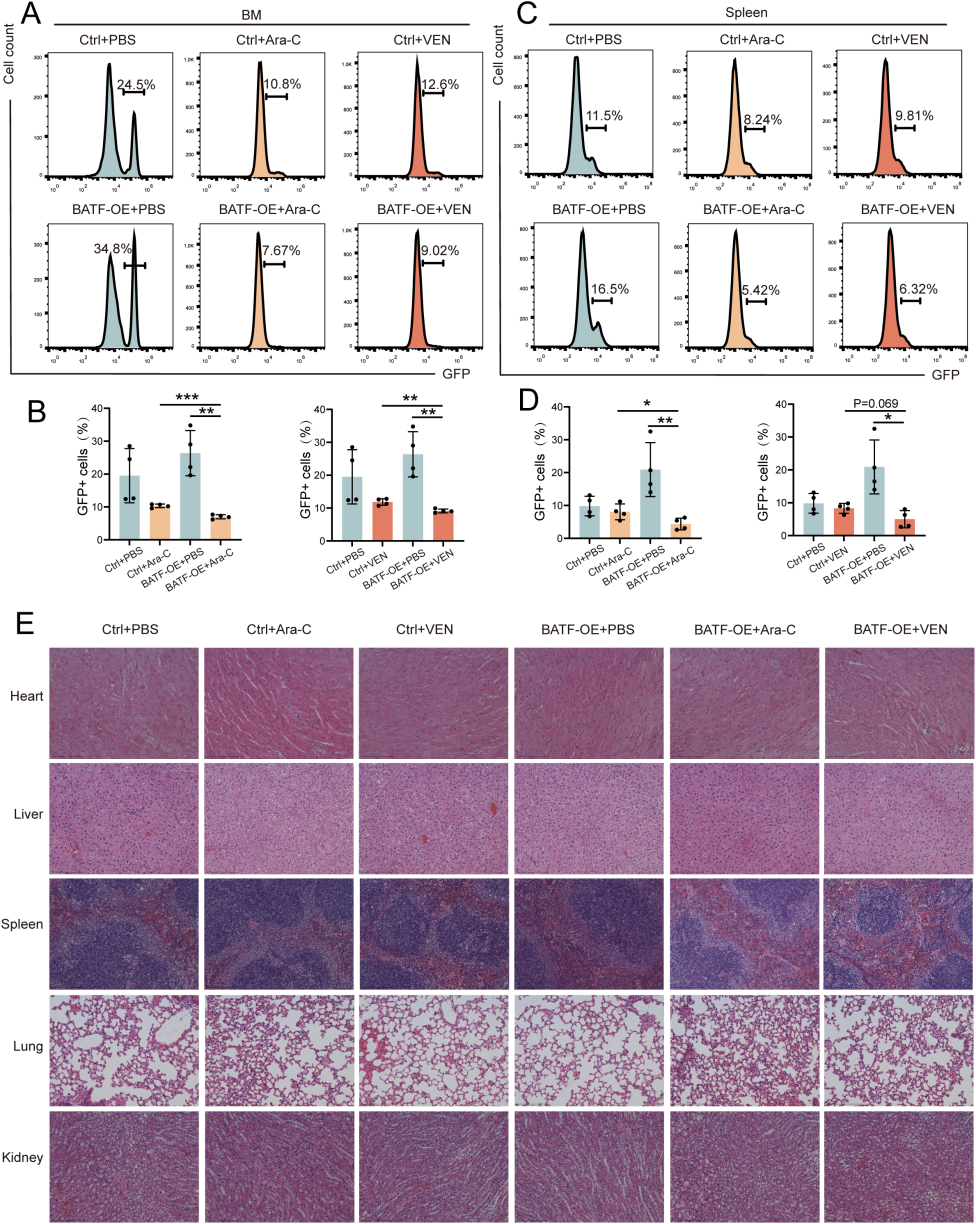
High expression of BATF enhance the drug sensitivity. **(A–D)** The proportion of GFP^+^ leukemia cells in the bone marrow and spleen analyzed in AML models. **(E)** Representative histopathological images of the major organs from AML mice. *P < 0.05, **P < 0.01, ***P < 0.001.

To further validate the effect of BATF expression on the sensitivity of chemotherapy drugs, we selected cytarabine and venetoclax, two commonly used first-line drugs in clinical practice, for *in vivo* validation. First, we established an AML mouse model by injecting C57BL/6 mice with GFP^+^ murine leukemia C1498 cells overexpressing BATF or control cells, followed by treatment with cytarabine and venetoclax. After sacrificing the mice, the results confirmed that the drug treatment sensitivity in the BATF overexpression group was higher than that in the control group, consistent with the findings from the bioinformatics analysis (**Figures 4A–D**). Additionally, we evaluated the side effects of BATF overexpression combined with chemotherapy on the mice. H&E staining showed no obvious signs of tissue damage in the heart, liver, spleen, lung, or kidneys of both control and BATF-overexpressing leukemic mice following treatment with cytarabine and venetoclax (**Figure 4E**). Our data collectively indicated that high expression of BATF could enhance the sensitivity to chemotherapeutic drugs, suggesting that it may serve as a potential therapeutic target for AML.

### 3.5 BATF is associated with the AML immune cell infiltration

**Figure 5:**
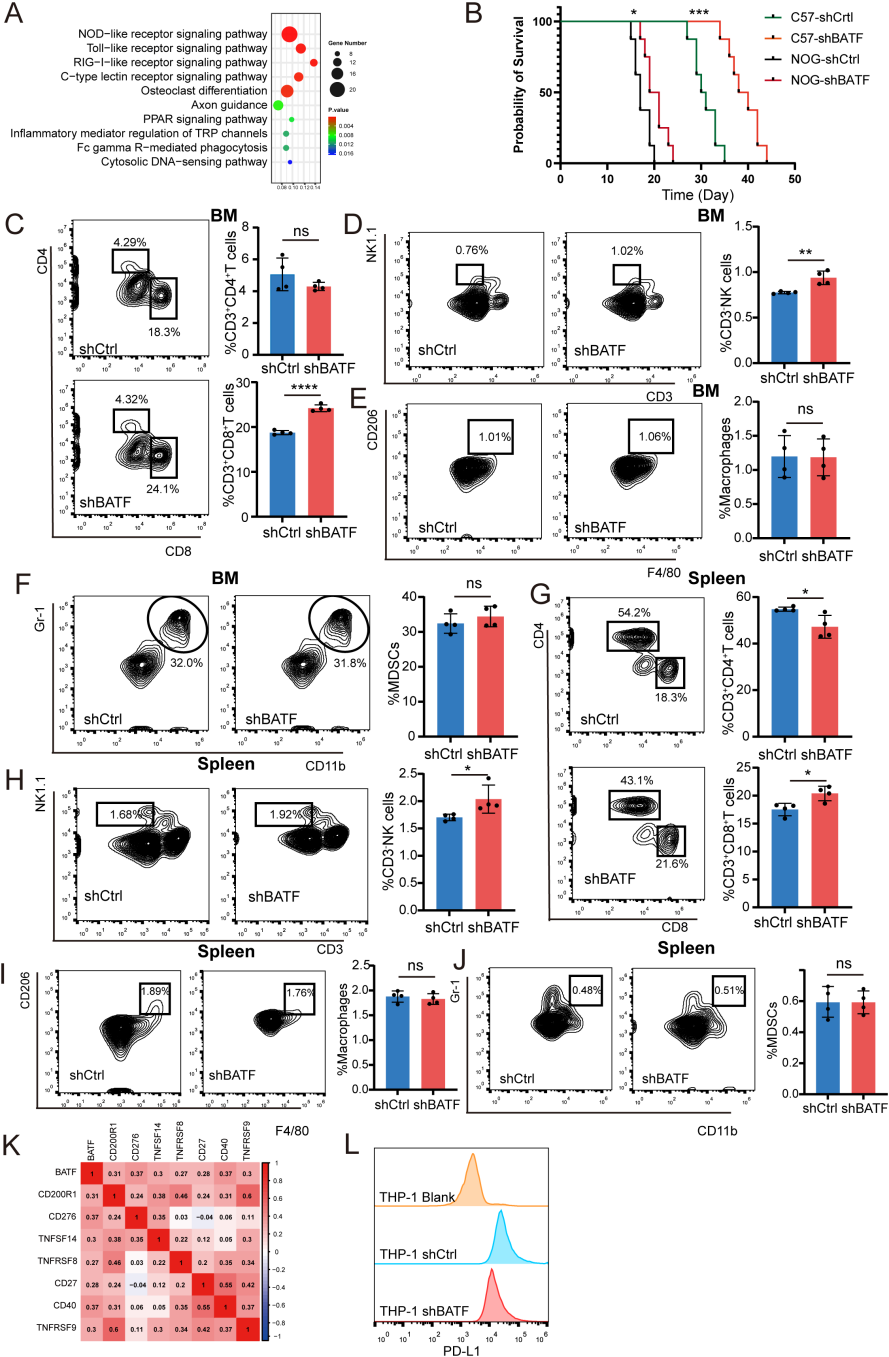
BATF expression is linked to the AML immune cell infiltration and immunotherapy efficacy. **(A)** ShBATF treatment and control C1498 cells were used for whole transcriptome RNA seq and subjected to KEGG enrichment analysis. **(B)** C57BL/6 or NOG mice (n = 8 per group) received intravenous injections of control or BATF knockdown C1498 cells, and their survival was monitored. **(C–F)** The proportion of T cells, NK cells, macrophages, and MDSC cells in the bone marrow of C1498 mouse models in the control group (n=4) and shBATF group (n=4). **(G–J)** The proportion of T cells, NK cells, macrophages, and MDSC cells in spleen of C1498 mouse models in the control group (n=4) and shBATF group (n=4). **(K)** Correlations between BATF expression and immune checkpoint expression. **(L)** Flow cytometry detection of PD-L1 expression in control cells and shBATF THP-1 cells. *P < 0.05, **P < 0.01, ***P < 0.001, ****P < 0.0001, ns, nonsignificant.

To further investigate the mechanism, we conducted RNA-seq in C1498 cells with either BATF knockdown or control and analyzed the results using the KEGG. The analysis revealed that BATF knockdown influenced pathways related to innate immune signaling (**Figure 5A**). To further validate the above conclusion, GFP-tagged C1498 cells with either BATF knockdown or control were injected into immunodeficient NOD.Cg-Prkdc scid Il2rg tm1Sug/JicCrl (NOG) mice and immunocompetent C57BL/6 mice. The results showed that the survival of mice in the BATF knockdown group was prolonged in both groups, but the extension was more significant in C57BL/6 mice compared to NOG mice (**Figure 5B**). This suggests that the impact of BATF expression on survival may be associated with the immune system. To examine the effects of BATF expression on immune cells, we analyzed main tumor microenvironment components via flow cytometry and identified that shBATF treated cells showed a significant increase in CD8^+^T cells and NK cells in the bone marrow compared to control cells (**Figures 5C, D**). However, the proportions of macrophages and myeloid-derived suppressor cells (MDSCs) in the bone marrow remained unchanged (**Figures 5E, F**). At the same time, the similar results were observed in the spleen (**Figures 5G–J**). Moreover, several immune checkpoint genes, including CD200R1, CD276, TNFSF14, TNFRSF8, CD27, CD40 and TNFRSF9, exhibited increased expression when BATF was highly expressed (**Figure 5K**). Finally, we examined PD-L1 expression in BATF-knockdown and control THP-1 cells using flow cytometry and found that BATF knockdown reduced PD-L1 expression (**Figure 5L**). In summary, these findings demonstrate that the antitumor effects triggered by BATF knockdown are predominantly reliant on CD8^+^T cells and NK cells. Our findings suggest that BATF expression is linked to the immune cell infiltration of AML.

### 3.6 The cell-cell communication of AML cells with BATF expression was explored

**Figure 6:**
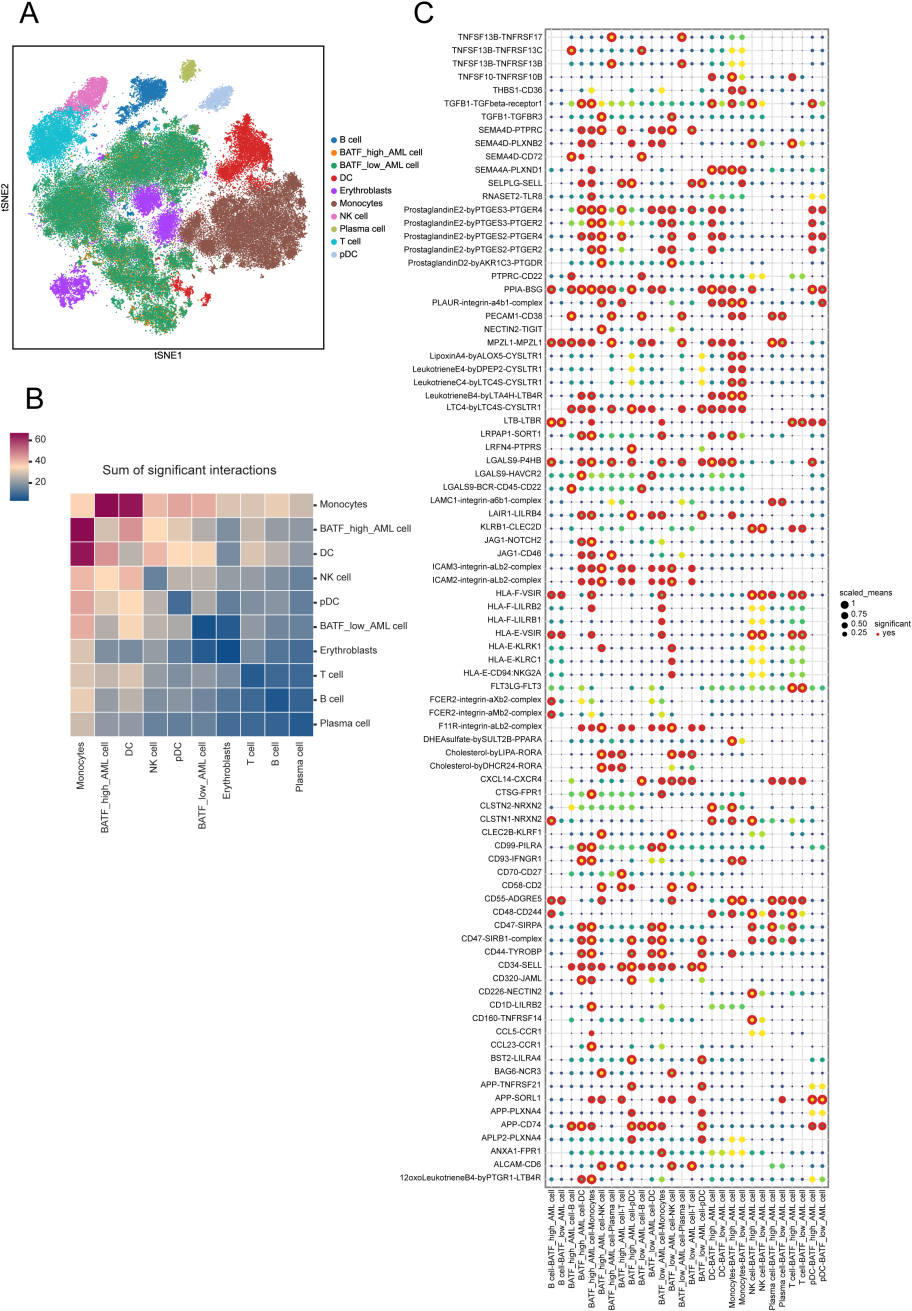
Cell interactions within the two clusters with varying BATF expression. **(A)** t-SNE for the dimension reduction and visualization of the ten cell types. **(B)** The number of significant interactions between various types of cells. **(C)** Visualization of receptor ligand pairs between AML cells with high and low expression of BATF and immune cells.

Exploring the impact of BATF on intercellular interactions may uncover new mechanisms through which BATF contributes to disease progression and therapy response. Therefore, we subsequently investigated the role of BATF in AML samples using single-cell sequencing analysis. Ten distinct cell types were identified in total (**Figure 6A**). The number of significant interactions between various types of cells is shown in **Figure 6B**. Analysis revealed significant differences in receptor ligand pairs between samples with high versus low BATF expression levels (**Figure 6C**). In summary, these findings suggest that BATF expression affects the communication between AML cells and other immune cells.

## 4 Discussion

AML is the most common aggressive haematologic malignancy associated with a very poor prognosis (21) and is characterised by poor leukocyte maturation and uncontrolled proliferation of haematopoietic stem cells. Although numerous medications, including targeted FLT3 inhibitors and IDH inhibitors, have been used for the treatment of AML (22), the majority of AML patients who receive chemotherapy relapse (23). Identifying the molecular mechanisms underlying AML progression, chemotherapeutic response, and relapse may have great clinical significance. Thus, the identification of novel biomarkers for diagnosing and treating AML holds significant importance.

The previous study has identified the expression and prognosis of BATF in AML, and we came to the same conclusion (24). We investigated whether BATF was highly expressed in AML patients through the TCGA database. Additionally, prognostic analysis showed that in AML patients, higher BATF mRNA expression was an independent predictor of OS and correlated with a shorter OS. Elevated BATF expression correlated with poorer overall survival across different age groups and cytogenetic risk categories, reinforcing its prognostic significance. We also performed ROC curve, univariate and multivariate analyses to determine whether BATF expression affects patient survival. Additionally, our results demonstrated that BATF expression promoted cell proliferation and was associated with shorter survival time in AML patients. These findings suggest the potential utilization of BATF as a prognostic indicator for patients with AML, thereby enhancing the prediction of their clinical outcomes.

It is known from previous literature that cancer progression is profoundly influenced by the immune microenvironment, which involves complex biological interactions (25). The bone marrow of AML patients has a significantly changed immunological environment, which exacerbates the disease (26). In previous research, researchers observed a difference between normal and AML BM immune cells. The relationship between immune infiltration and AML prognosis was investigated by using a single-cell RNA sequencing dataset from previous research (27). In our study, previous results showed that the survival of mice in the BATF knockdown group was prolonged in both groups, but the extension was more significant in C57BL/6 mice compared to NOG mice, suggesting that the impact of BATF on survival may be mediated by the immune system. Further analysis using RNA-seq and KEGG pathways revealed a correlation between BATF and immune signaling pathways. Additionally, flow cytometry was performed to assess the effect of BATF expression on immune cell proportions, and the results showed that BATF knockdown increased the proportions of CD8^+^ T cells and NK cells, thereby supporting the above conclusion. Moreover, we found that targeting BATF could potentially enhance the efficacy of AML immunotherapy through bioinformatics analysis and experimental validation. Gene expression analysis of peripheral blood CD8^+^ T cells from AML patients highlighting the pivotal role of CD8^+^ T cells in AML progression (28). Research using murine AML models has demonstrated that as the disease advances, the proportion of CD8^+^ T cells expressing multiple inhibitory receptors (IRs) increases. However, their cytotoxic activity can be restored through checkpoint inhibition or genetic deletion, thereby enhancing antileukemia immune responses (29, 30). In the present study, research and development regarding oncology were primarily driven by cancer immunology and immunotherapy, with an emphasis on CD8^+^ T-cells and the tumour microenvironment (31). Meanwhile, immunotherapy utilizing NK cells represents a promising approach for cellular therapy in AML, particularly in cases of refractory or relapsed disease, and offers potential applications across various stages of AML progression (32–34). These results suggested that there were variations in immune cell infiltration between TME patients with high and low BATF expression.

Moreover, we investigated the relationship between BATF expression and drug sensitivity and found that the IC50s of AZD5582, dabrafenib, ibrutinib, cytarabine, gemcitabine, temozolomide and venetoclax were significantly lower in the high-expression group, while that of dasatinib was greater in the high-expression group. To further validate, we selected cytarabine and venetoclax for *in vivo* experiments and found that BATF overexpression enhanced drug sensitivity and improved therapeutic efficacy, consistent with our previous conclusions. The findings suggested that the expression level of BATF dramatically affected the effect of chemotherapy in patients with AML, providing new insights into precision therapy for AML patients.

Furthermore, single-cell sequencing analysis revealed AML cells with varying levels of BATF expression within the tumor microenvironment. Our results showed there were differences in receptor ligand pairs between AML cells with high and low expression of BATF and immune cells, which could provide assistance for further exploration of the mechanism.

Despite being built on high-quality analyses and thorough experiments, our study has certain limitations that should be acknowledged. Firstly, our study was limited by a relatively small sample size, which may affect the generalizability of the results. Then, the mechanisms underlying the phenomenon have not been explored in sufficient depth and require further investigation.

Collectively, our bioinformatics and experimental analyses systematically revealed relationships between BATF expression and the level of immune cell infiltration and the prognosis of AML patients. To further our understanding of the functional role of BATF in AML processes, we conducted a comprehensive functional pathway analysis, drawing upon both experimental and bioinformatics approaches to gain deeper insights into its mechanisms.

## 5 Conclusion

In conclusion, our bioinformatics analysis and experimental research results suggest that BATF expression could alter the AML immune cell infiltration and may function as a potential therapeutic target for AML. Further fundamental research on the specific downstream mechanisms of BATF in AML progression still needs to be conducted.

## Data Availability

The original contributions presented in the study are included in the article/**Supplementary Material**. Further inquiries can be directed to the corresponding authors.
