# Supplementary material for: Identification and validation of BATF as a prognostic biomarker and regulator of immune cell infiltration in acute myeloid leukemia
Source: Front Immunol. 2025 Jan 13;15:1429855. doi: 10.3389/fimmu.2024.1429855 (PMC11769954; doi:10.3389/fimmu.2024.1429855)
Supplement: Supplementary file 1 [file Table1.docx]

**Identification and validation of BATF as a prognostic biomarker and regulator of immune cell infiltration in acute myeloid leukemia**

Table S1. Primer Sequence for RT-qPCR.

| Gene (Human) | Forward | Reverse |
| --- | --- | --- |
| *GAPDH* | GGAGCGAGATCCCTCCAAAAT | GGCTGTTGTCATACTTCTCATGG |
| *BATF* | TATTGCCGCCCAGAAGAGC | GCTTGATCTCCTTGCGTAGAG |
| *PMVK* | CCTTTCGGAAGGACATGATCC | TCTCCGTGTGTCACTCACCA |
| *PSMD8* | GCCGTAAATCAGGCGGTCT | GCCCTTGAGTTGCTCGTACA |
| *TCTA* | GTGGTTGGTGTTAAGTCTCCTG | GGAAATGCGTGGAGCCATCA |
| *TINF2* | GTGGAACATTTTCCGCGAGTA | GCCCATACAAAGGCGTTCG |

Table S2. shRNA Sequence for BATF.

|  | The Sense Strand | The Antisense Strand |
| --- | --- | --- |
| sh#1 | 5′CCGGGAGAAACAGAACGCGGCTCTACTCGAGTAGAGCCGCGTTCTGTTTCTCTTTTTG3′ | 5′AATTCAAAAAGAGAAACAGAACGCGGCTCTACTCGAGTAGAGCCGCGTTCTGTTTCTC3′ |
| sh#2 | 5′CCGGCCACGCATTCCACCAACCTCACTCGAGTGAGGTTGGTGGAATGCGTGGTTTTTG3′ | 5′AATTCAAAAACCACGCATTCCACCAACCTCACTCGAGTGAGGTTGGTGGAATGCGTGG3′ |


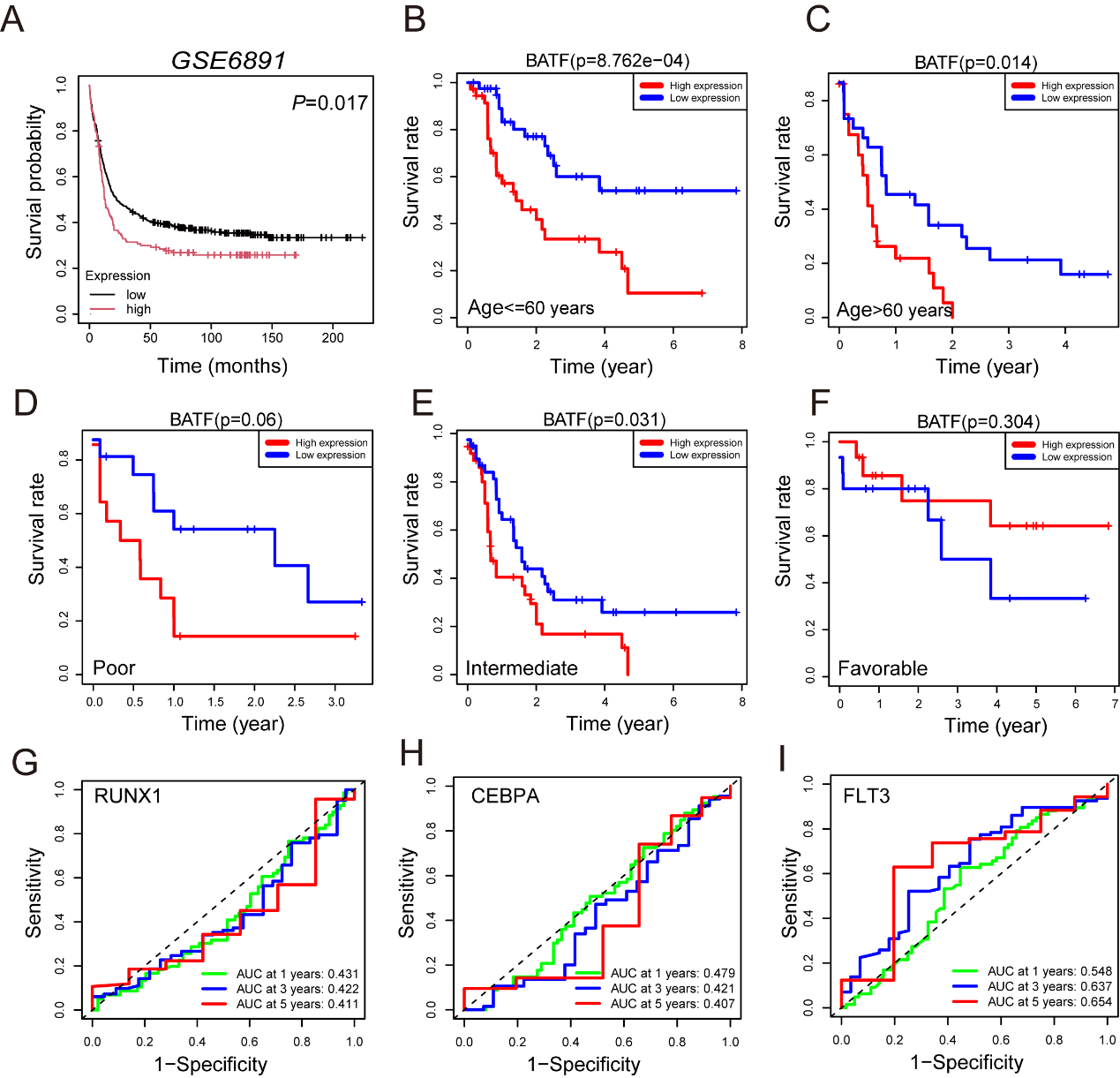


**Figure S1** Prognosis significance of BATF of AML patients. **(A)** KM survival analysis of BATF in the GSE6891 database. **(B, C)** KM survival analysis between BATF expression and age. **(D-F)** KM survival analysis between BATF expression and ELN risk group. **(G-I)** The AUC of RUNX1, CEBPA and FLT3 in the TCGA database.


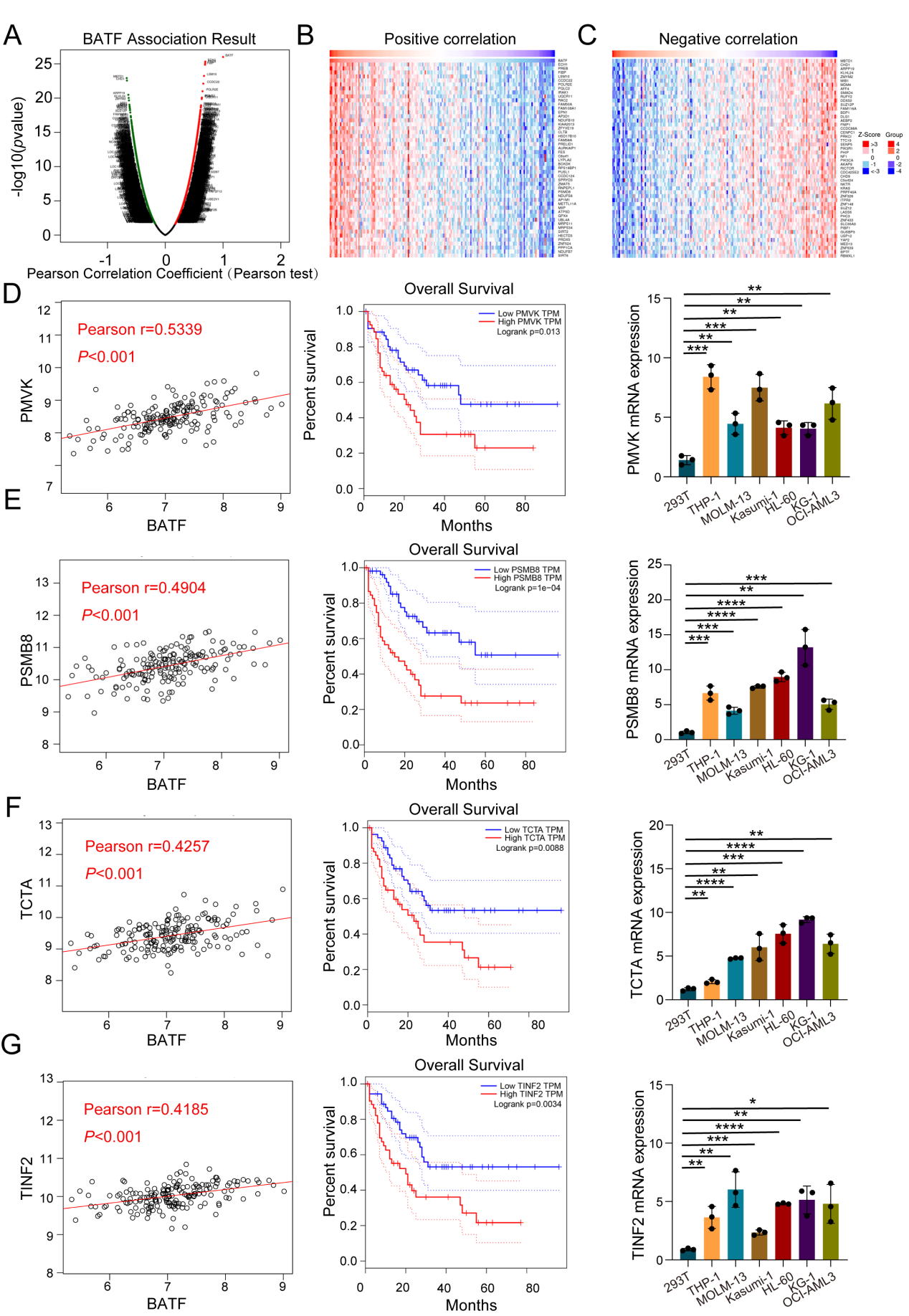


**Figure S2** The expression levels of genes associated with BATF are significantly elevated and inversely associated with overall survival. **(A)** The genes associated with BATF expression are displayed in the volcano map. **(B, C)** Heatmaps showing genes with differential expression that are positively and negatively regulated, respectively. **(D-G)** Scatter plots showing the Pearson correlation between BATF expression, the prognostic significance and the expression patterns of these four genes in AML patients.

**
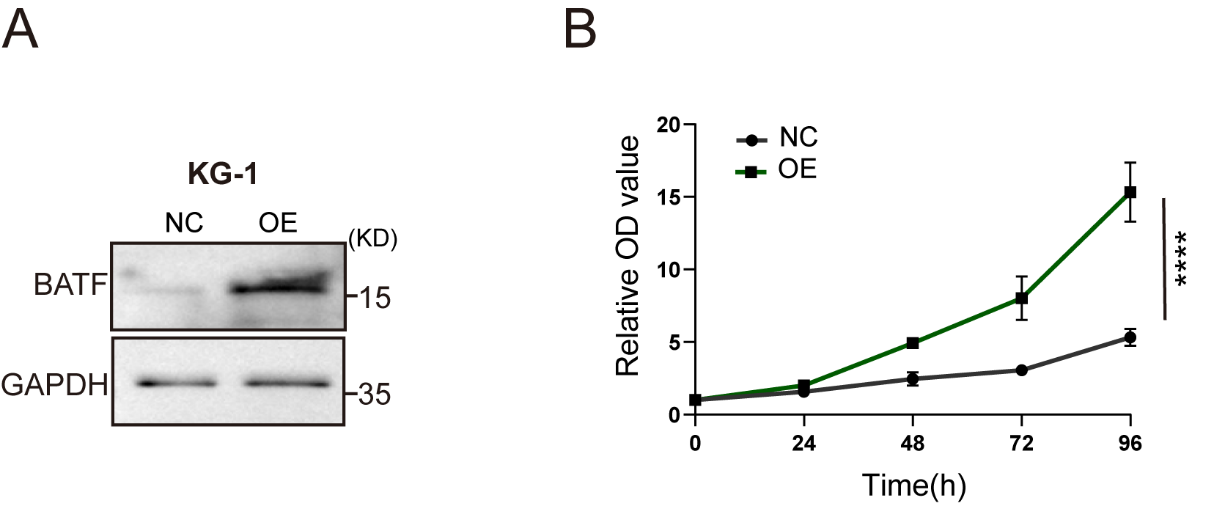
**

**Figure S3** BATF expression stimulates the growth and multiplication of KG-1 cells. **(A)** KG-1 cells were transduced with BATF-expressing lentivirus or control lentivirus. The overexpression efficacy of BATF was determined by Western blotting. **(B)** CCK-8 assay of BATF-overexpressing and control cells among the KG-1 cells.
